# Supplementary material for: Assessment of the Polyphenolic Profile and Beneficial Effects of Red and Green Propolis in Skin Inflammatory Conditions and Oxidative Stress
Source: Biomedicines. 2025 Sep 10;13(9):2229. doi: 10.3390/biomedicines13092229 (PMC12466955; doi:10.3390/biomedicines13092229)
Supplement: Supplementary file 1 [file biomedicines-13-02229-s001.zip › biomedicines-3807599-supplementary.pdf]

biomedicines

MDPI

**Supplementary**

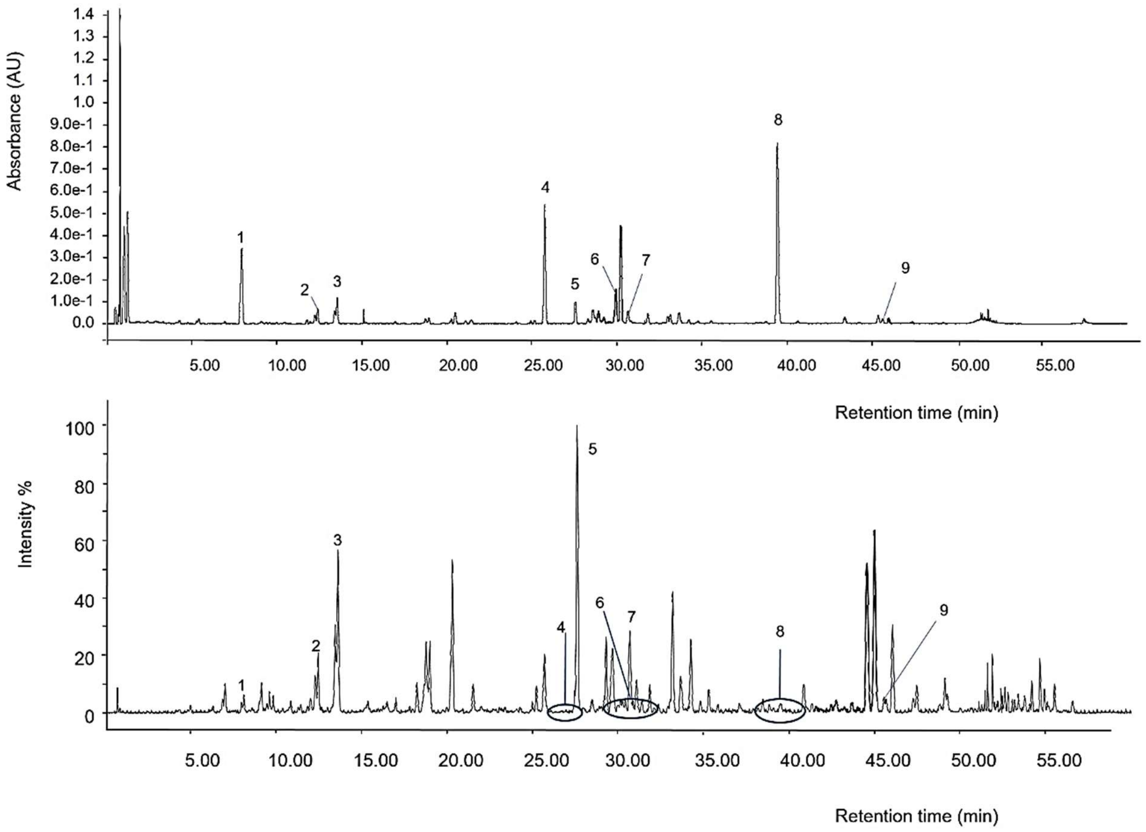


**Figure S1.** Comparison of HPLC-UV/PDA and MS/TOF chromatograms of green propolis. Numbers indicate the principal peaks identified by spectroscopic and spectrophotometric parameters.

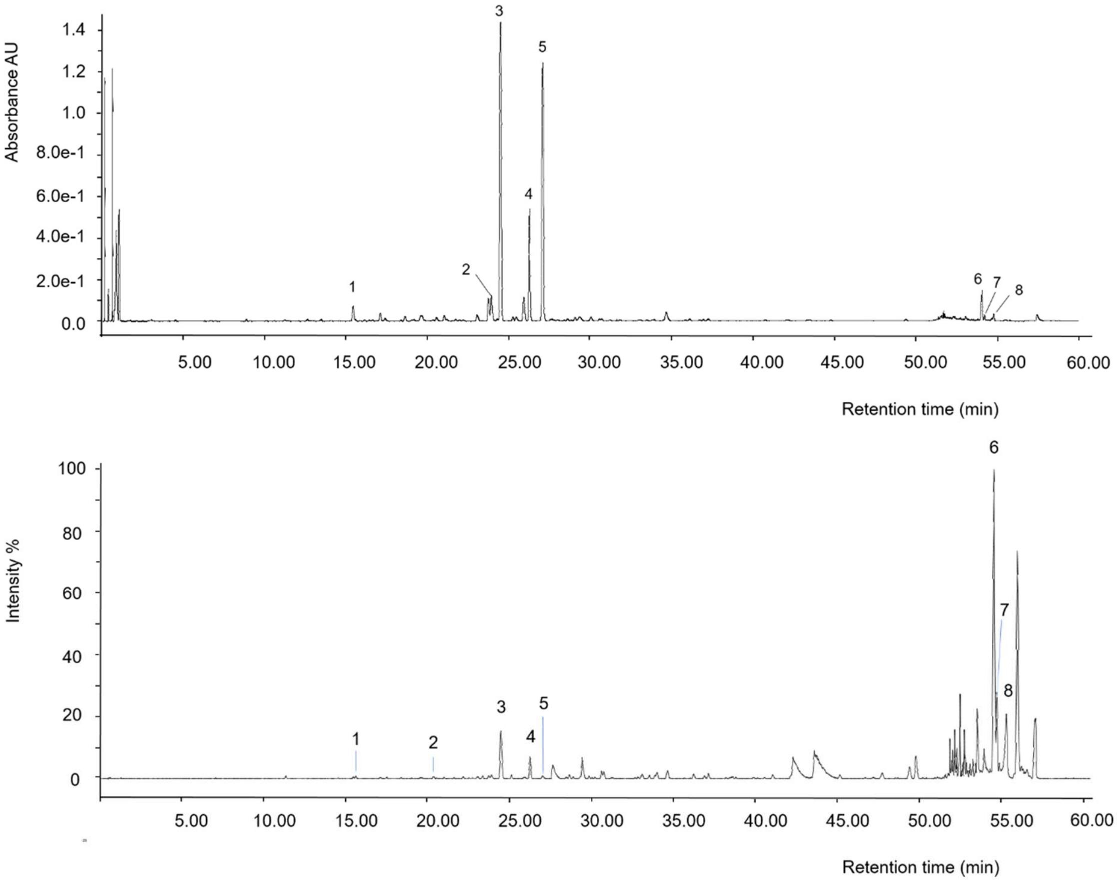


**Figure S2.** Comparison of HPLC-UV/PDA and MS/TOF chromatograms of red propolis. Numbers indicate the principal peaks identified by spectroscopic and spectrophotometric parameters.

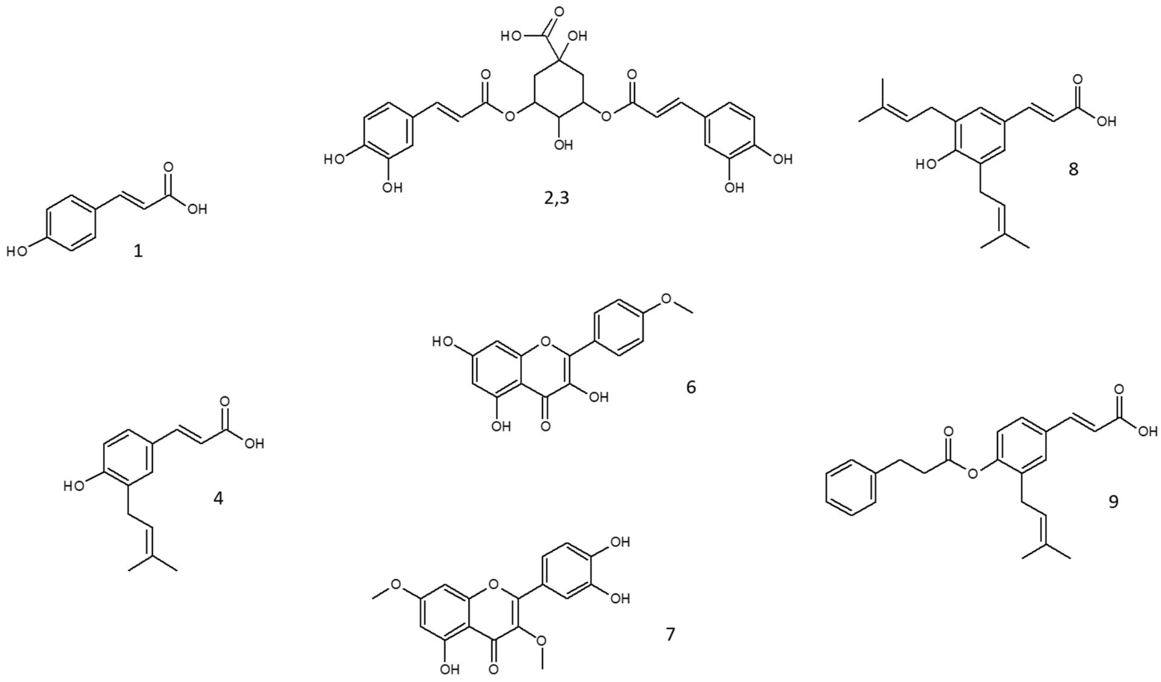

**Figure S3.** Structures of the main compounds identified in green propolis through HPLC-ESI-MS: *p*-coumaric acid (**1**), di-O-caffeoylquinic acid (**2**, **3**), drupanin (**4**), kaempferide (**6**), dimethylquercetin (**7**), artepillin C (**8**), baccharin (**9**).

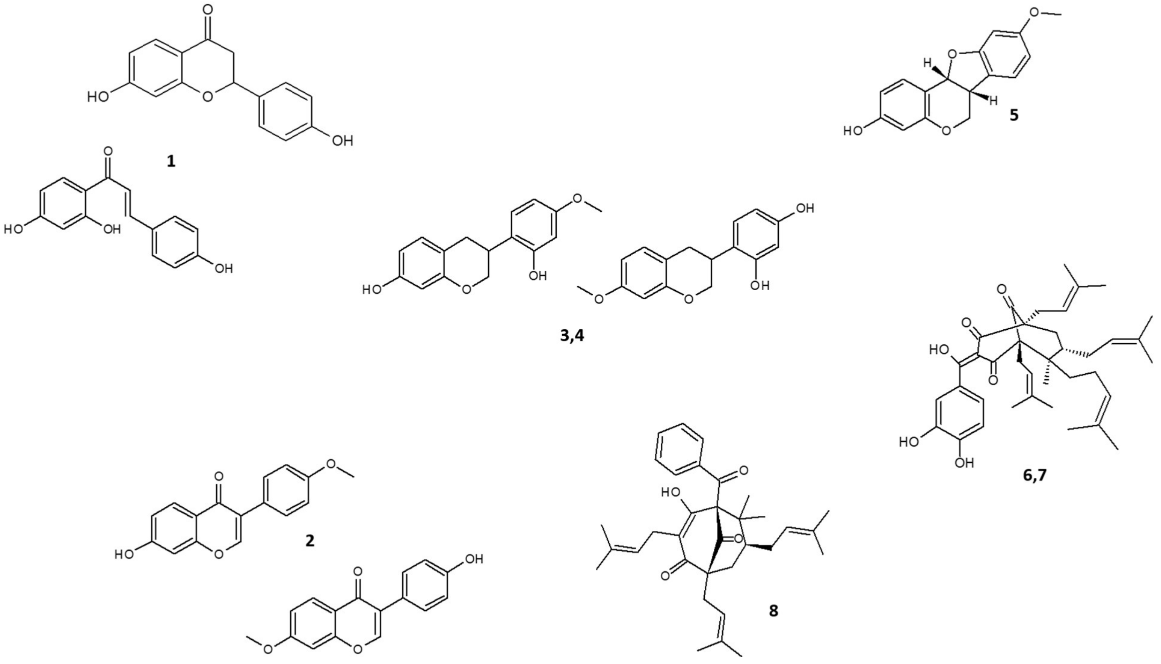

**Figure S4.** Structures of the main compounds identified in red propolis through HPLC-ESI-MS: liquiritigenin/isoliquiritigenin (**1**), formononetin/isoformononetin (**2**), vestitol/neovesitol (**3**, **4**), medicarpin (**5**), guttiferone (**6**, **7**), nemorosone (**8**).

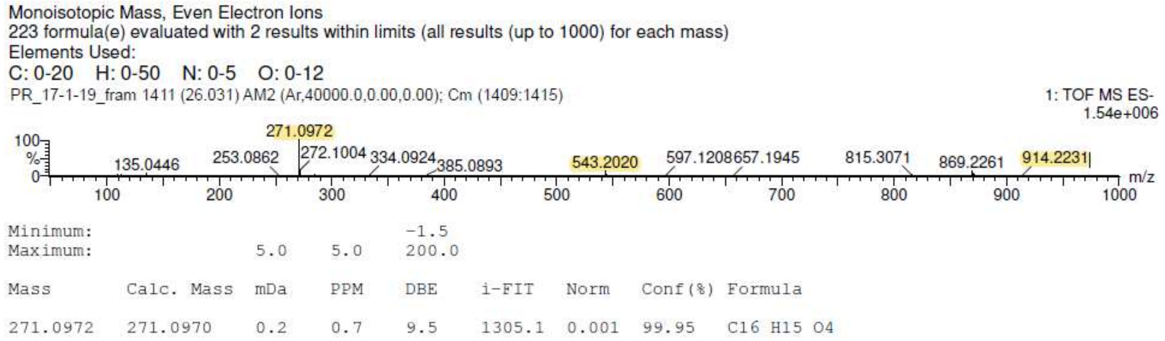


**Figure S5.** TOF mass spectrum of neovestitol/vestitol. The molecular peak has *m/z* s 271.0972 [M-H]$^{-}$, the dimer peak has *m/z* 543.2020 [M-H]$^{-}$.

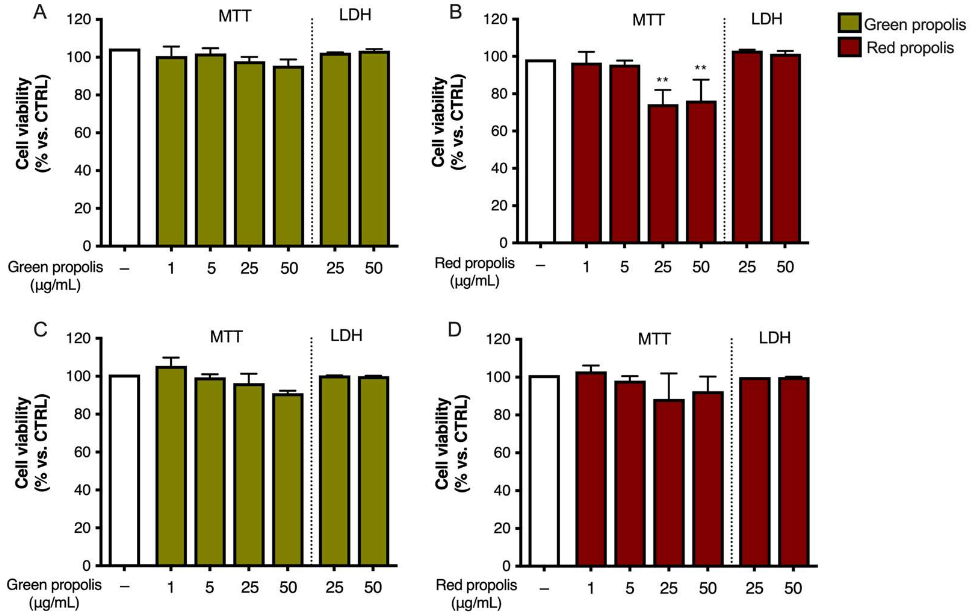


**Figure S6.** Assessment of green and red Brazilian propolis effect on HaCaT (**A**, **B**) and HDF (**C**, **D**) cell viability through MTT and LDH assays. Cells were treated for 6 h in presence of increasing propolis concentrations. Data are expressed as a percentage of the control, which was arbitrarily assigned the value of 100%. ** $p < 0.01$ versus control.

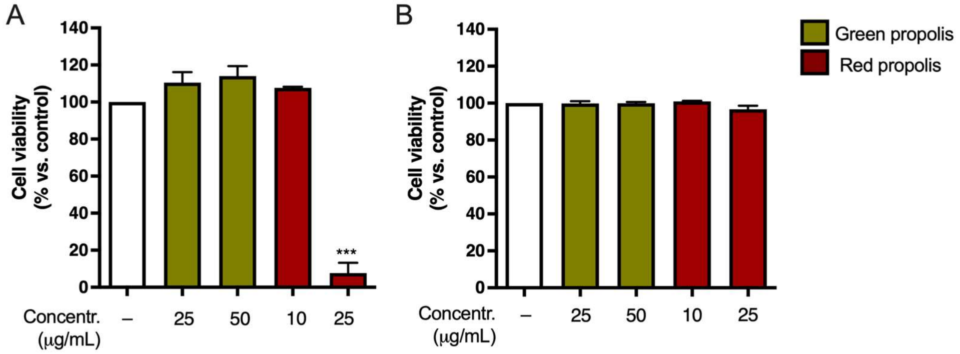


**Figure S7.** Assessment of green and red Brazilian propolis effect on HaCaT (**A**) and HDF (**B**) cell viability through LDH assay. Cells were treated for 24 h in presence of increasing propolis

concentrations. Data are expressed as a percentage of the control, which was arbitrarily assigned the value of 100%. *** $p < 0.001$ versus control.

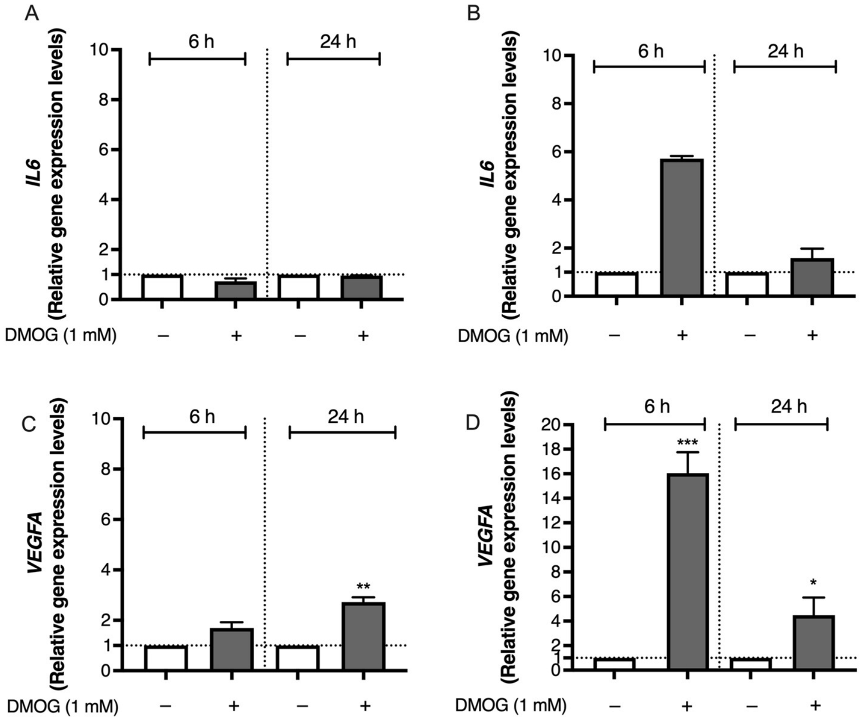


**Figure S8.** Gene expression analysis of *IL6* (**A**, **B**) and *VEGFA* (**C**, **D**) in HaCaT (**A**, **C**) and HDF (**B**, **D**) cells treated for 6 or 24 h with DMOG (1 mM). * $p < 0.05$, ** $p < 0.01$, *** $p < 0.001$ versus control.

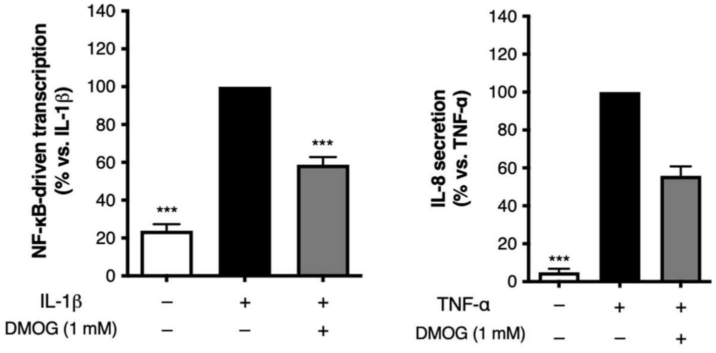


**Figure S9.** Assessment of DMOG effect on NF-κB-driven transcription in HaCaT cells. Cells were stimulated with 10 ng/mL of IL-1β and treated for 6 h with DMOG (1 mM). Data are expressed as a percentage of the stimulus, which was arbitrarily assigned the value of 100%. *** $p < 0.001$ versus IL-1β.
